# Effect of traditional plants in Sri Lanka on skin keratinocyte count

**DOI:** 10.1016/j.dib.2018.03.044

**Published:** 2018-03-15

**Authors:** Katsura Sano, Takao Someya, Kotaro Hara, Yoshimasa Sagane, Toshihiro Watanabe, R.G.S. Wijesekara

**Affiliations:** a ALBION Co. Ltd., 1-7-10 Ginza, Chuo-ku, Tokyo 104-0061, Japan; b Department of food and Cosmetic Science, Faculty of Bioindustry, Tokyo University of Agriculture, 196 Yasaka, Abashiri, Hokkaido 099-2493, Japan; c Department of Aquaculture and Fisheries, Faculty of Livestock, Fisheries and Nutrition, Wayamba University of Sri Lanka, Makandura, Gonawila 60170, Sri Lanka

**Keywords:** Cell number, Keratinocytes, Calcein assay, Traditional plant, Medical herb

## Abstract

This article describes the effects of extracts of several plants collected in Sri Lanka on the number of human skin keratinocytes. This study especially focuses on the plants traditionally used in indigenous systems of medicine in Sri Lanka, such as Ayurveda, as described below (English name, “local name in Sri Lanka,” scientific name). Neem plant,”kohomba,” *Azadirachta indica* (Sujarwo et al., 2016; Nature’s Beauty Creations Ltd., 2014) [1,2], emblic myrobalan plant, “nelli,” *Phyllanthus emblica* (Singh et al., 2011; Nature’s Beauty Creations Ltd., 2014) [3,4], malabar nut plant, “adhatoda,” *Justicia adhatoda* (Claeson et al., 2000; Nature’s Beauty Creations Ltd., 2014) [5,6], holy basil plant, “maduruthala,” *Ocimum tenuiflorum* ( Cohen et al., 2014; Nature’s Beauty Creations Ltd., 2014) [7,8]. The expression profiles are provided as line graphs.

**Specifications Table**

**Table:** 

Subject area	*Biology*
More specific subject area	*Cell biology*
Type of data	*Graph*
How data was acquired	*Fluorescent microscope (SpectraMax® i3x, MOLECULAR DEVICES)*
Data format	*Analyzed*
Experimental factors	*Cell number*
Experimental features	*Analysis of cell number by calcein assay*
Data source location	*Negombo, Sri Lanka*
Data accessibility	*Data are available within this article*

**Value of the data**

• Data represent changes in keratinocyte numbers after exposure to several plant extracts.
• These data indicate that several plant extracts regulate keratinocyte numbers in the epidermis, and could be further investigated as pharmacologic and cosmetic agents.

## 1 Data

This data article contains line graphs showing the effects of extracts of several plants collected in Negombo, Sri Lanka, on the number of human skin keratinocytes. Cells were treated with various concentrations (0–0.3%) of each plant extracts for 24 h, and percent cell viability was calculated relative to that of untreated controls. Data represent the mean ± SE values from triplicate independent experiments (*P<0.05, **P<0.001 and ***P<0.001 vs. control).

## 2 Experimental design, materials and methods

All plants were collected from a medicinal garden at the Institute of Traditional Plants in Sri Lanka (Negombo, Sri Lanka). Fresh leaves of neem plant (”kohomba,” *Azadirachta indica*) [1], [2] were extracted with 15 times its weight of 50% BG at room temperature for 24 h. Fresh leaves of emblic myrobalan plant (“nelli,” *Phyllanthus emblica*) [3], [4] were extracted with 7.5 times its weight of 70% EtOH at room temperature for 24 h. Fresh leaves of malabar nut plant (“adhatoda,” *Justicia adhatoda*) [5], [6] were extracted with 5 times its weight of 70% EtOH at room temperature for 24 h. Fresh aerial parts of holy basil plant (“maduruthala,” *Ocimum tenuiflorum*) [7], [8] were extracted with 3 times its weight of 70% EtOH at room temperature for 24 h.

### 2.1 Keratinocyte cell culture

Normal human epidermal keratinocytes (HEKn; GIBCO) were isolated from neonatal foreskin. The cells were cultured in Medium 154 (Invitrogen) supplemented with human keratinocyte growth factor (HKGS; Invitrogen), according to the manufacturer's instructions. Cells were grown at 37 °C in a humidified incubator containing 5% CO_2_. For all of the experiments, human keratinocytes were seeded (3×10^3 cells/well) in a 96-well plate, and incubated for 8 h with culture media containing HKGS. The cells were next subjected to HKGS starvation for 16 h with Medium 154.

### 2.2 Cell number analysis (calcein assay)

To determine cell viability, cells were seeded (3×10^3 cells/well) in a 96-well plate. Cells were exposed to various concentrations of plant extracts for 24 h. The cells were then stained with 10 mM calcein-AM (Dojindo) in the dark for 30 min at 37 °C and washed with phosphate-buffered saline (PBS). The fluorescence intensity (em/ex, 485/530 nm) of each well was measured using a SpectraMax® i3x fluorescence microplate reader (MOLECULAR DEVICES). Data were calculated as the percent cell viability compared to that of controls without plants extracts treatment and have been presented as the mean and SE values for triplicate wells.

### 2.3 Statistical analysis

**Fig. 1:**
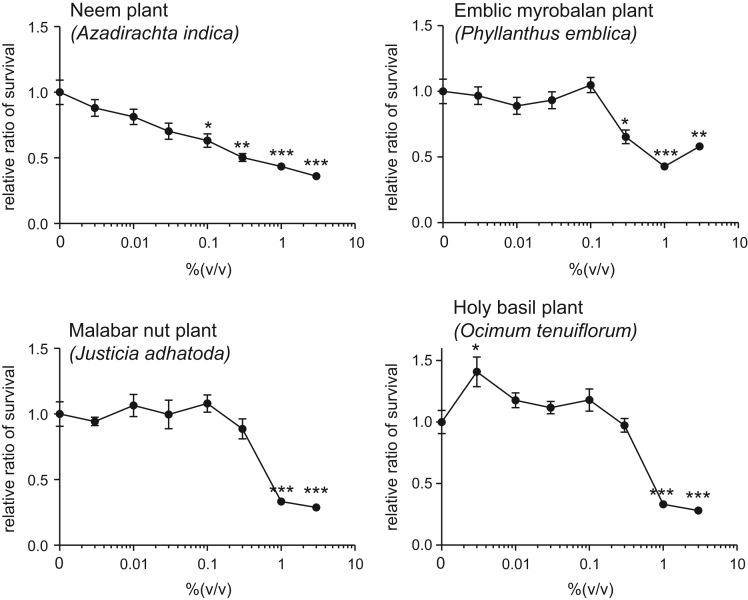
Cell viability of HEKn detected by calcein assay. Cells were treated with various concentrations (0–0.3%) of each plant extracts for 24 h, and percent cell viability was calculated relative to that of untreated controls. The values are shown as the mean±SE of three independent experiments.

All the values have been reported in terms of mean±SE values. The data were analyzed using the Student's *t*-test. A *P* value less than 0.05 was considered to be statistically significant (Fig. 1).
